# Racial and Urban-Rural Difference in the Frequency of Ischemic Stroke as Initial Manifestation of Atrial Fibrillation

**DOI:** 10.3389/fpubh.2021.780185

**Published:** 2021-11-05

**Authors:** Jingchuan Guo, Nico Gabriel, Jared W. Magnani, Utibe R. Essien, Walid F. Gellad, Maria M. Brooks, Ludovic Trinquart, Emelia J. Benjamin, Inmaculada Hernandez

**Affiliations:** ^1^Center for Pharmaceutical Prescribing and Policy, University of Pittsburgh, Pittsburgh, PA, United States; ^2^Department of Pharmaceutical Outcomes and Policy, University of Florida, Gainesville, FL, United States; ^3^Center for Drug Evaluation and Safety, University of Florida, Gainesville, FL, United States; ^4^Division of Clinical Pharmacy, University of California, San Diego, San Diego, CA, United States; ^5^Department of Medicine, University of Pittsburgh, Pittsburgh, PA, United States; ^6^Department of Epidemiology, University of Pittsburgh, Pittsburgh, PA, United States; ^7^Department of Biostatistics, Boston University School of Public Health, Boston, MA, United States; ^8^Department of Medicine, Boston University School of Medicine, Boston, MA, United States

**Keywords:** atrial fibrillation, ischemic stroke, racial differences, urban/rural, Medicare claims

## Abstract

**Objective:** Atrial fibrillation (AF) may remain undiagnosed until the development of complications. We aimed to examine the epidemiology and racial/ethnic and rural/urban differences in the frequency of newly diagnosed AF manifesting as ischemic stroke in a nationally representative sample of Medicare beneficiaries.

**Methods:** We used a 5% random sample of Medicare claims to identify patients newly diagnosed with AF in 2016. The primary dependent variable was stroke or transient ischemic attack (TIA) in the 7 days *prior* to the first AF diagnosis, i.e., stroke or TIA as the initial manifestation of AF. We constructed a multivariable logistic regression to quantify the association between race/ethnicity, urban/rural residence, and the primary dependent variable.

**Results:** Among 39,409 patients newly diagnosed with AF (mean age 77 ± 10 years; 58% women; 7.2% Black, 87.8% White, 5.1% others), 2,819 (7.2%) had ischemic stroke or TIA in the 7 days *prior* to AF diagnosis. Black patients (adjusted OR [95% CI]: 1.21 [1.05, 1.40], vs. White) and urban residents (1.21 [1.08, 1.35], vs. rural) were at increased risk of stroke as the initial manifestation of AF. Racial differences were larger among patients aged ≥75 years, with adjusted ORs of 1.43 (1.19, 1.73) for Black vs. White patients, but non-significant for those aged <75 (*P* for interaction = 0.03).

**Conclusion:** We observed significant and important differences in the risk of stroke as initial manifestation of AF between White and Black patients and between rural and urban residents. Our results suggest potential disparities in the identification AF across race/ethnicity groups and urban/rural areas.

## Introduction

Atrial fibrillation (AF) is a strong risk factor for ischemic stroke and is implicated in 15–20% of stroke cases (1). Preventive treatment with oral anticoagulation may reduce stroke risk by two-thirds in patients with AF (2). However, AF can be asymptomatic and remain undiagnosed until the development of complications such as stroke.

Some studies have examined the prevalence of stroke as the initial manifestation of AF but results vary substantially (3–7). The Framingham Heart Study reported that 3.4% of incident AF patients had a stroke or transient ischemic attack (TIA) in the month *prior* to AF detection (3, 5). In a cohort of 3,507 patients newly diagnosed with AF at the University of Pennsylvania system, Patel et al. estimated that 5.3% of patients had a stroke in the week *prior* to AF diagnosis (7). However, these community- and health system- based cohorts may have limited generalizability to the general population of patients newly diagnosed with AF across the US.

In the present study, we aimed to examine the epidemiology of ischemic stroke or TIA occurring in the seven days *prior* to the first AF diagnosis, i.e., stroke or TIA as the initial manifestation of AF, using a nationally representative sample of Medicare beneficiaries. Given the previously recognized health disparities in the awareness of AF (8), we also measured racial/ethnic and rural/urban differences in the frequency of incident AF manifesting as ischemic stroke or TIA.

## Methods

This retrospective cohort study used claims data from a 5% random sample of Medicare Part D beneficiaries obtained from the Centers for Medicare and Medicaid Services (CMS). Using the CMS Chronic Condition Data Warehouse indicator of AF, we identified beneficiaries who were first diagnosed with AF between January 1, 2016, and December 31, 2016. We constrained sampling to those who were continuously enrolled in Medicare Part D in the year *prior* to AF diagnosis (i.e., index date). This ensured that we had access to complete medical information at baseline to make certain that the sample represented incident AF patients. The final sample included 39,409 beneficiaries newly diagnosed with AF in 2016.

The dependent variable was ischemic stroke or TIA as the initial manifestation of AF. We defined this outcome as one claim with a primary or secondary International Classification of Diseases, Ninth Revision (ICD-9) for stroke or TIA based on diagnostic code of 430, 431, 433.x1, 434.x1, 435, or 436 or an ICD-10 diagnostic code of G45 or I63 on the day of or within seven days *prior* to AF diagnosis. The 7 day cutoff was selected based on prior literature that a 7-day period captured majority of prior-AF stroke (7). In identifying stroke events, we used provider claims (carrier claims) instead of institutional claims to be able to capture with higher accuracy the date of the first stroke diagnosis: Inpatient (institutional) claims are often billed on the day of discharge and include codes for primary and secondary diagnoses identified during a hospitalization, which would prevent us from accurately depicting the timing between first AF diagnosis and stroke, if they both happened within a hospitalization. In contrast, claims for physician services provided during an inpatient hospitalization (provider claims) are billed with the date of provision of service, which enabled us to estimate the relative timing between first AF diagnosis and stroke diagnosis with a higher accuracy.

The exposures of interest included race/ethnicity and urban vs. rural residence, which were measured on the index date. We collected self-reported race/ethnicity information from Medicare's administrative files and categorized individuals into three groups: non-Hispanic White, non-Hispanic Black, and other races/ethnicities (hereafter denoted as White, Black, and others). We classified participants as living in an urban or rural area using zip codes and nine-level United States Department of Agriculture (USDA) Rural-Urban Continuum Codes. Consistent with the USDA classification, we defined urban patients as those living in levels 1–3 and rural patients as those living in levels 4–9 (9).

In addition to race/ethnicity and urban vs. rural residence, we also examined information concerning other demographics and clinical characteristics, including age, sex, residence in one of four Census regions (Northeast, Midwest, South, and West), CHA_2_DS_2_-VASc score, valvular disease, eligibility for Medicaid, receipt of low-income subsidy, end-stage renal disease, and area deprivation index (ADI). A patient's CHA_2_DS_2_-VASc score was calculated based on age, sex, history of stroke/TIA, vascular disease, heart failure, hypertension, and diabetes.

In the cohort of newly diagnosed AF, we computed age- and sex-adjusted proportion (%) of stroke or TIA as initial manifestation of AF by race/ethnicity and urban/rural area. We then constructed a multivariable logistic regression to quantify the association between race/ethnicity and urban/rural residence and the risk of stroke or TIA as initial manifestation of AF, while adjusting for demographic and clinical characteristics, as listed above. We tested the interaction effects of race/ethnicity and urban/rural residence with age (<75 years vs. ≥75 years), sex, and ADI (<90^th^ percentile vs. ≥90^th^ percentile) in the multivariable-adjusted model. A two-sided p <0.05 was considered significant, and analyses were performed using SAS v9.4 (SAS Institute, Cary, NC).

## Results

Among 39,409 patients who were newly diagnosed with AF, 2,819 (7.2%) had ischemic stroke or TIA in seven days *prior* to AF diagnosis. Mean age of the study cohort was 77 (standard deviation [SD], 10) years; over half (58%) were female, and 80% were urban residents. Compared to White beneficiaries (87.8%) with AF, Black beneficiaries (7.2%) and beneficiaries of other races/ethnicities (5.1%) were younger, more likely to reside in urban areas, and more likely to be eligible for Medicaid coverage and low-income subsidy. Black beneficiaries had higher CHA_2_DS_2_-VASc scores and were more likely to live in the South and in areas with higher ADIs than White beneficiaries or beneficiaries of other races/ethnicities (Supplementary Table 1).

**Figure 1:**
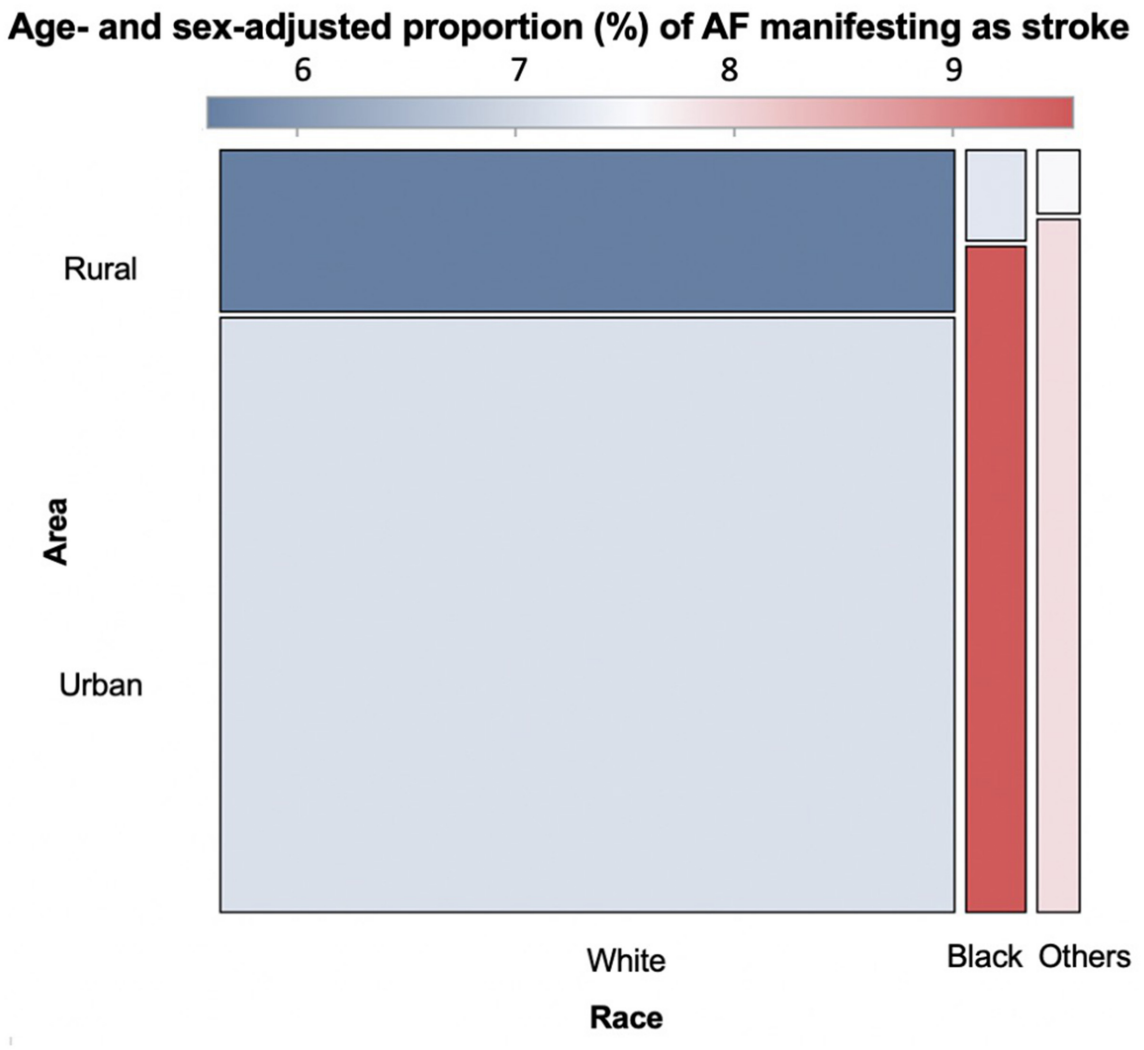
Age- and sex-adjusted proportion (%) of newly diagnosed atrial fibrillation patients with ischemic stroke as the first manifestation of atrial fibrillation by race and urban/rural residence. The x axis shows three race/ethnicity categories (White, Black and Others) and the y axis shows urban and rural areas. Each of the 6 boxes presents one race-area group (White-Urban, White-Rural, Black-Urban, Black-Rural, Others-Urban, and Others-Rural). The size of the box represents the sample size of each group. Dark blue indicates the lowest proportion of AF diagnosis manifesting as stroke, while dark red represents the highest proportion of AF diagnosis manifesting as stroke.

In our cohort of newly diagnosed AF, in urban areas, the age- and sex-adjusted proportion of stroke or TIA occurring 7 days *prior* to AF diagnosis was 7.1% (95% CI: 6.8, 7.5) for White beneficiaries, 9.6% (8.4, 10.8) for Black beneficiaries, and 7.9% (6.8, 9.3) for beneficiaries of other races/ethnicities. In rural areas, the age- and sex-adjusted proportion of stroke or TIA occurring 7 days *prior* to AF diagnosis was 5.6% (5.1, 6.1) for White beneficiaries, 7.2% (4.9, 10.6) for Black beneficiaries, and 7.6% (4.4, 12.9) for beneficiaries of other races/ethnicities (Figure 1).

After adjusting for covariates, Black beneficiaries (adjusted OR [95% CI]: 1.21 [1.05, 1.40]) were at significantly increased risk of having stroke or TIA as initial AF manifestation compared to White beneficiaries (Table 1). The risk of stroke or TIA as initial AF manifestation did not significantly differ between White patients and patients of other races/ethnicities (adjusted OR: 1.16 [0.97, 1.40]). Urban residents were 21% more likely (adjusted OR: 1.21 [1.08, 1.35]) to have stroke or TIA as initial AF manifestation compared to rural residents.

**Table 1:** Adjusted odds ratios of new AF diagnosis manifesting as stroke across racial/ethnicity groups and between urban and rural areas.

		**Stroke cases% (*n*)**	**Adjusted odds ratio (95%CI)**
Race/ethnicity	White	7.0% (*n =* 2,406)	Reference
	Black	9.1% (*n =* 256)	1.22 (1.06, 1.41)
	Others	7.8% (*n =* 157)	1.16 (0.97, 1.40)
Areas	Urban	7.5% (*n =* 2,366)	Reference
	Rural	5.7% (*n =* 453)	1.21 (1.08, 1.35)

*Adjusted for age, sex, CHA_2_DS_2_-VAS_c_ score, valvular disease, geographic region (Northeast, South, Midwest, and West), Medicaid enrollment, low income subsidy, end stage renal disease, and area deprivation index*.

**Figure 2:**
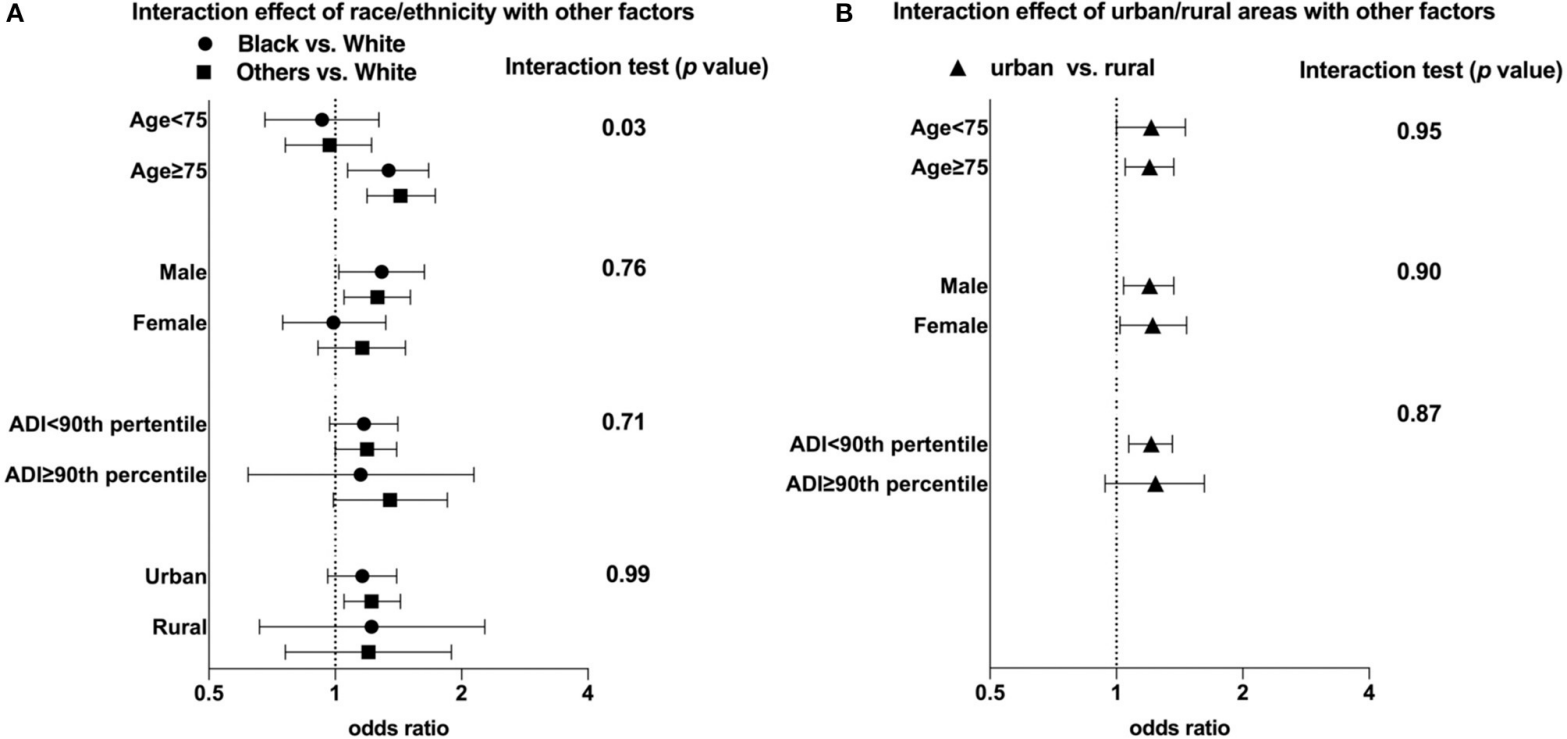
Interactions of race/ethnicity and urban/rural residence with age, sex, and area of deprivation index. ADI, Area of Deprivation Index. In addition to the listed factors, the model was also adjusted for CHA_2_DS_2_-VAS_c_ score, valvular disease, geographic region (Northeast, South, Midwest, and West), Medicaid enrollment, low income subsidy and end stage renal disease.

We observed a significant interaction between race/ethnicity and age (*p* = 0.03). The increased risk of stroke or TIA as initial AF manifestation observed for Black patients compared to White was statistically significant among those aged 75 years or older (adjusted OR: 1.43 [1.19, 1.73], vs. White) but not in those <75 years (adjusted OR: 0.97 [0.76, 1.22]; p_interaction_ = 0.03); Figure 2.

## Discussion

Our study measured the risk of stroke as an initial manifestation of AF in a nationally representative sample of Medicare beneficiaries. We observed that over 7% of Medicare beneficiaries newly diagnosed with AF presented with ischemic stroke or TIA seven or fewer days prior to AF diagnosis. After adjusting for relevant factors, Black race and urban residency were associated with increased risk of stroke or TIA as initial AF manifestation.

The Framingham Heart Study reported a lower frequency of stroke or TIA as an initial manifestation of AF (3.4%) than that estimated in the present study (3); even with the inclusion of stroke cases occurring up to 30 days *prior* to AF diagnosis in Framingham. The Framingham Heart Study report included participants with incident AF diagnosed between 1951 and 2013, whereas the current study reflects the more contemporary era with much more widespread use of telemetry in stroke patients. The secular trends of post-stroke telemetry use over the decades may explain the different estimates between two studies. Differences in the demographic characteristics of study populations may have also contributed to the diverging estimates, since Framingham participants were largely of European ancestry, whereas our Medicare sample is more diverse in terms of race/ethnicity, region of residence, and socioeconomic status. Our results are comparable with data from the Penn Atrial Fibrillation Free study, which estimated that 5.4% of patients newly diagnosed with AF 2004–2009 had a stroke in the 7 days prior to AF diagnosis (7). Quantifying strokes attributable to undiagnosed AF is important because potentially they could be preventable with oral anticoagulation, at least among individuals who would be eligible for anticoagulation, which represents 96% of our sample given CHA_2_DS_2_-VASc scores ≥2 in men or ≥3 in women.

Large-scale screening for AF has been widely discussed as a potential public health strategy for early detection and treatment of AF that can help to prevent stroke and other AF-associated complications (10). However, there remain several major concerns, including the identification of target population for screening, its cost-effectiveness, the selection of screening devices, and the development of intervention strategies for detected AF cases (11). In this context, the availability of generalizable estimates of stroke as the initial manifestation of AF is paramount to producing robust estimates of the cost-effectiveness of potential AF screening strategies and identifying populations who may benefit the most from screening.

Racial differences in stroke incidence and death have been reported frequently in the general population, with these differences decreasing with older age (12). Similar to the Penn Atrial Fibrillation Free study (7), we observed that Black beneficiaries had a 22% higher risk of having stroke or TIA as initial AF manifestation compared to White beneficiaries. We further observed that such racial differences were larger among patients first diagnosed with AF at an age of ≥75 years. There is increasing awareness that race is a social construct (13). Whereas the pathophysiology of racial differences is uncertain; instead of reflecting biological variation, racial differences in the manifestation of AF may reflect structural inequities including racism, decreased awareness of AF, lower access to care, residential environment, and inequitable control of underlying AF and stroke risk factors.

Although prior work has suggested that rural residents often face barriers to healthcare access in general (14), we observed a lower risk of incident AF manifesting as stroke or TIA in rural residents than in urban residents across all three racial/ethnic subgroups. Similar to our findings, prior data from Medicare claims revealed a greater risk of stroke in urban areas in the year of AF diagnosis (15). Further studies are needed to address the underlying causes of the these observed differences between White and Black patients and between urban and rural residents.

Several limitations of the present study should be noted. First, because some patients may have had fatal strokes or had unrecognized AF, it is possible that our results underestimate the proportion of patients who had an ischemic stroke as fist AF manifestation. On the other hand, it is possible that in some patients, AF occurred as a consequence of the stroke event. Second, we created a binary classification of urban vs. rural residence. We acknowledge that urban and rural are not homogeneous categories; future work might further distinguish urban, suburban, exurban, and rural areas. Last, we acknowledge that the race/ethnicity category “Others” is heterogenous; we did not examine Hispanics and Asian subgroups separately because of a lack of power.

In conclusion, in our cohort of Medicare beneficiaries with AF, we observed a clinically relevant proportion having ischemic stroke or TIA occurring within seven days prior to AF diagnosis. Black patients and urban residents were more likely to present with stroke or TIA as initial AF manifestation. Further research is needed to evaluate the effectiveness of AF screening efforts in reducing inequities in health outcomes associated with AF.

## Data Availability Statement

Our Medicare Claims data was obtained from CMS. Requests to access these datasets should be directed to resdac@umn.edu.

## Author Contributions

JG designed the study, executed the disparities analyses, and drafted the first draft of the manuscript. NG contributed to the study design and executed the data management and statistical analyses. JM, UE, WG, MB, LT, and EB contributed to the generation of the study question, study design, and critical review of the manuscript. IH supervised the project and was responsible for obtaining funding, coordinating research efforts, and supervising analyses and manuscript writing. All authors contributed to the article and approved the submitted version.

## Funding

Research reported in this study was supported by the National Heart, Lung and Blood Institute (IH, Grant No. K01HL142847). EB was supported by R01HL092577; American Heart Association AF 18SFRN34110082. JM was supported by R01HL143010 and R33HL144669.

## Conflict of Interest

IH reports scientific advisory board fees from Pfizer and Bristol Myers Squibb, outside of the submitted work. The remaining authors declare that the research was conducted in the absence of any commercial or financial relationships that could be construed as a potential conflict of interest.

## Publisher's Note

All claims expressed in this article are solely those of the authors and do not necessarily represent those of their affiliated organizations, or those of the publisher, the editors and the reviewers. Any product that may be evaluated in this article, or claim that may be made by its manufacturer, is not guaranteed or endorsed by the publisher.

## References

[1] Ferro JM. Atrial fibrillation and cardioembolic stroke. Minerva Cardioangiol. (2004) 52:111–24. 15194993

[2] Crystal E Connolly SJ. Role of oral anticoagulation in management of atrial fibrillation. Heart. (2004) 90:813–7. 10.1136/hrt.2003.021642 15201261 PMC1768341

[3] Lubitz SA Yin X McManus DD Weng L-C Aparicio HJ Walkey AJ. Stroke as the initial manifestation of atrial fibrillation. Stroke. (2017) 48:490–2. 10.1161/STROKEAHA.116.015071 28082669 PMC5262530

[4] Jaakkola J Mustonen P Kiviniemi T Hartikainen JEK Palomäki A Hartikainen P. Stroke as the first manifestation of Atrial Fibrillation. PLoS ONE. (2016) 11:1–9. 10.1371/journal.pone.0168010 27936187 PMC5148080

[5] Lin HJ Wolf PA Benjamin EJ Belanger AJ D'agostino RB. Newly diagnosed atrial fibrillation and acute stroke: The framingham study. Stroke. (1995) 26:1527–30. 7660392 10.1161/01.str.26.9.1527

[6] Borowsky LH Regan S Chang Y Ayres A Greenberg SM Singer DE. First diagnosis of atrial fibrillation at the time of stroke. Cerebrovasc Dis. (2017) 43:192–9. 10.1159/000457809 28208140

[7] Patel PJ Katz R Borovskiy Y Killian A Levine JM McNaughton NW. Race and stroke in an atrial fibrillation inception cohort: findings from the penn atrial fibrillation free study. Hear Rhythm. (2018) 15:487–93. 10.1016/j.hrthm.2017.11.025 29475795 PMC5879006

[8] Meschia JF Merrill P Soliman EZ Howard VJ Barrett KM Zakai NA. Racial disparities in awareness and treatment of atrial fibrillation: The REasons for geographic and racial differences in stroke (REGARDS) study. Stroke. (2010) 41:581–7. 10.1161/STROKEAHA.109.573907 20190000 PMC2885129

[9] United States Department of Agriculture Economic Research Service. USDA ERS - Rural-Urban Commuting Area Codes. Available online at: https://www.ers.usda.gov/data-products/rural-urban-commuting-area-codes/ (accessed June 22, 2019)

[10] Schnabel RB Haeusler KG Healey JS Freedman B Boriani G Brachmann J. Searching for atrial fibrillation poststroke: a white paper of the AF-SCREEN international collaboration. Circulation. (2019) 140:1834–50. 10.1161/CIRCULATIONAHA.119.040267 31765261

[11] Benjamin EJ Go AS Desvigne-Nickens P Anderson CD Casadei B Chen LY. Research priorities in atrial fibrillation screening: a report from a national heart, lung, and blood institute virtual workshop. Circulation. (2021) 143:372–88. 10.1161/CIRCULATIONAHA.120.047633 33493033 PMC8776506

[12] Howard G Moy CS Howard VJ McClure LA Kleindorfer DO Kissela BM. Where to focus efforts to reduce the black-white disparity in stroke mortality: incidence versus case fatality? Stroke. (2016) 47:1893–8. 10.1161/STROKEAHA.115.012631 27256672 PMC4927373

[13] Churchwell K Elkind MSV Benjamin RM Carson AP Chang EK Lawrence W. Call to action: structural racism as a fundamental driver of health disparities: a presidential advisory from the american heart association. Circulation. (2020) 142:454–68. 10.1161/CIR.0000000000000936 33170755

[14] Harrington RA Califf RM Balamurugan A Brown N Benjamin RM Braund WE. Call to action: Rural health: a presidential advisory from the american heart association and american stroke association. Circulation. (2020) 141:E615–44. 10.1161/CIR.0000000000000753 32078375

[15] Guo J He M Magnani JW Brooks MM Gellad WF Hernandez I. Comparison of oral anticoagulant use and stroke risk among older adults newly-diagnosed atrial fibrillation living in urban-versus-rural areas. Am J Cardiol. (2020) 130:64–9. 10.1016/j.amjcard.2020.06.015 32680675 PMC8040970

