# Supplementary material for: Racial and Urban-Rural Difference in the Frequency of Ischemic Stroke as Initial Manifestation of Atrial Fibrillation
Source: Front Public Health. 2021 Nov 5;9:780185. doi: 10.3389/fpubh.2021.780185 (PMC8602106; doi:10.3389/fpubh.2021.780185)
Supplement: Supplementary file 1 [file Data_Sheet_1.PDF]

Supplement

Title: Racial and Urban-Rural Disparities in the Frequency of Ischemic Stroke as First Manifestation of Atrial Fibrillation

Author list: Jingchuan Guo, Nico Gabriel, Jared W Magnani, Utibe R. Essien, Walid F. Gellad, Maria M Brooks, Ludovic Trinquart, Emelia J. Benjamin, Inmaculada Hernandez

Table S1 Baseline characteristics by race/ethnicity

Table S2. Results of multivariable logistic regression model.

Figure S1. Sex-adjusted proportion (%) of newly diagnosed atrial fibrillation patients with ischemic stroke as the first manifestation by race and age.

Table S1 Baseline characteristics by race/ethnicity

Variable, n (%)	White	Black	Others
Male	14543 (42.1)	1150 (40.8)	982 (49.0)
Age, ≥75 years	21371 (61.8)	1351 (47.9)	1096 (54.6)
Urban residents	27198 (78.6)	2485 (88.1)	1836 (91.5)
Region			
Northeast	7196 (20.8)	448 (15.9)	374 (18.6)
Midwest	9278 (26.8)	590 (20.9)	235 (11.7)
South	12972 (37.5)	1586 (56.2)	536 (26.7)
West	5136 (14.9)	197 (7.0)	861 (42.9)
Area deprivation index ≥90th percentile	2941 (8.5)	730 (25.9)	178 (8.9)
Receipt of low-income subsidy	8191 (23.7)	1824 (64.7)	1319 (65.8)
Eligible for Medicaid coverage	5030 (14.5)	1161 (41.2)	1008 (50.2)
$CHA_2DS_2\text{-}VAS_c$ score			
0-2	3104 (9.0)	185 (6.6)	215 (10.7)
3-4	11625 (33.6)	756 (26.8)	592 (29.5)
5+	19853 (57.4)	1880 (66.6)	1199 (59.8)
Valvular disease	785 (2.3)	52 (1.8)	34 (1.7)
End stage renal disease	809 (2.3)	398 (14.1)	161 (8.0)

Table S2. Results of multivariable logistic regression model.

Covariates	Odds ratio	95% CI	
Male vs. Female	1.24	1.14	1.35
Age ≥75 vs. <75 years	0.67	0.61	0.73
Black vs. White	1.22	1.06	1.41
Others vs. White	1.16	0.97	1.40
Urban vs. Rural	1.21	1.08	1.35
Region			
Northeast vs South	1.02	0.92	1.13
Midwest vs South	0.99	0.89	1.09
West vs South	1.03	0.91	1.16
Area deprivation index ≥ 90th vs. <90th	0.98	0.85	1.12
Receipt of low-income subsidy	1.07	0.95	1.21
Eligible for Medicaid enrollment	0.73	0.63	0.85
CHA_2DS_2-VAS_c score			
3-4 vs. 0-2	2.54	1.77	3.65
5+ vs. 0-2	19.00	13.41	26.93
Valvular disease	0.84	0.65	1.09
End stage renal disease	0.51	0.40	0.65

Figure S1. Sex-adjusted proportion (%) of newly diagnosed atrial fibrillation patients with ischemic stroke as the first manifestation by race and age.

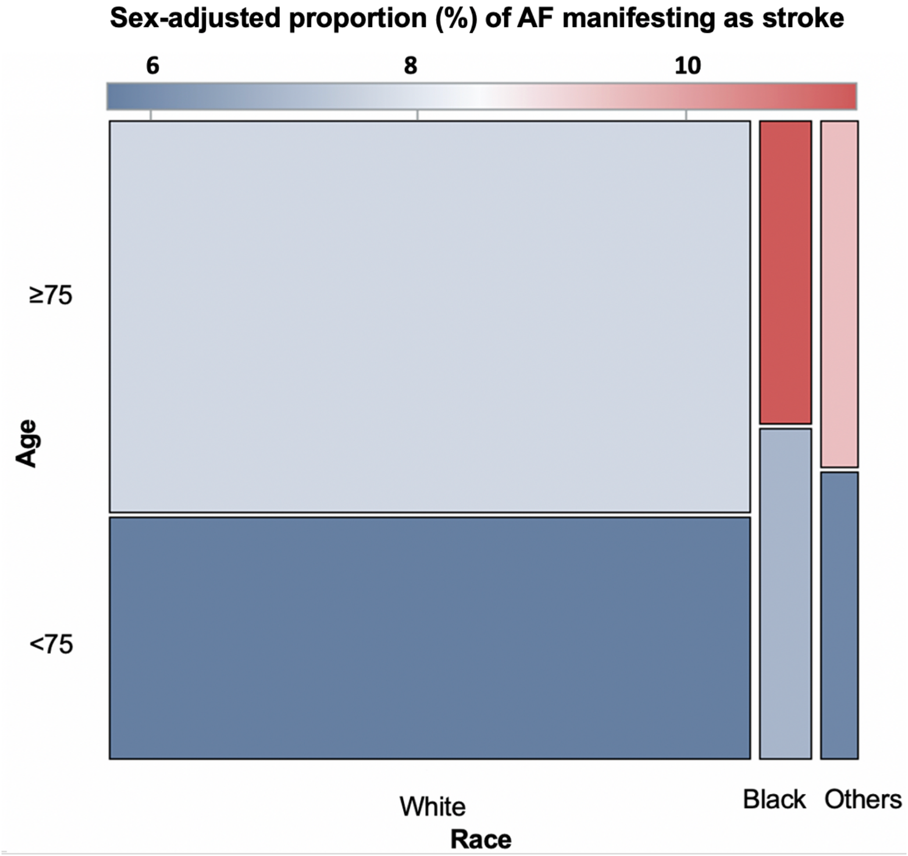

The x axis shows three race/ethnicity categories (White, Black and Others) and the y axis shows two age groups: age ≥ vs. ≤ 75 years of age. Each of the 6 boxes presents one race-age group (White-age<75, White-age≥75, Black-age<75, Black-age≥75, Others-age<75, and Others-age≥75). The size of the box represents the sample size of each group. Dark blue indicates the lowest proportion of AF diagnosis manifesting as stroke, while dark red represents the highest proportion of AF diagnosis manifesting as stroke.
